# Co‐occurrence dynamics of endangered Lower Keys marsh rabbits and free‐ranging domestic cats: Prey responses to an exotic predator removal program

**DOI:** 10.1002/ece3.3954

**Published:** 2018-03-26

**Authors:** Michael V. Cove, Beth Gardner, Theodore R. Simons, Allan F. O'Connell

**Affiliations:** ^1^ NC Cooperative Fish and Wildlife Research Unit Department of Applied Ecology North Carolina State University Raleigh NC USA; ^2^ School of Environmental and Forest Science University of Washington Seattle WA USA; ^3^ U.S. Geological Survey NC Cooperative Fish and Wildlife Research Unit Department of Applied Ecology North Carolina State University Raleigh NC USA; ^4^ U.S. Geological Survey Patuxent Wildlife Research Center Laurel MD USA

**Keywords:** invasive species, occupancy, predator management, predator–prey dynamics

## Abstract

The Lower Keys marsh rabbit (*Sylvilagus palustris hefneri*) is one of many endangered endemic species of the Florida Keys. The main threats are habitat loss and fragmentation from sea‐level rise, development, and habitat succession. Exotic predators such as free‐ranging domestic cats (*Felis catus*) pose an additional threat to these endangered small mammals. Management strategies have focused on habitat restoration and exotic predator control. However, the effectiveness of predator removal and the effects of anthropogenic habitat modifications and restoration have not been evaluated. Between 2013 and 2015, we used camera traps to survey marsh rabbits and free‐ranging cats at 84 sites in the National Key Deer Refuge, Big Pine Key, Florida, USA. We used dynamic occupancy models to determine factors associated with marsh rabbit occurrence, colonization, extinction, and the co‐occurrence of marsh rabbits and cats during a period of predator removal. Rabbit occurrence was positively related to freshwater habitat and patch size, but was negatively related to the number of individual cats detected at each site. Furthermore, marsh rabbit colonization was negatively associated with relative increases in the number of individual cats at each site between survey years. Cat occurrence was negatively associated with increasing distance from human developments. The probability of cat site extinction was positively related to a 2‐year trapping effort, indicating that predator removal reduced the cat population. Dynamic co‐occurrence models suggested that cats and marsh rabbits co‐occur less frequently than expected under random conditions, whereas co‐detections were site and survey‐specific. Rabbit site extinction and colonization were not strongly conditional on cat presence, but corresponded with a negative association. Our results suggest that while rabbits can colonize and persist at sites where cats occur, it is the number of individual cats at a site that more strongly influences rabbit occupancy and colonization. These findings indicate that continued predator management would likely benefit endangered small mammals as they recolonize restored habitats.

## 1 INTRODUCTION

**Figure 1:**
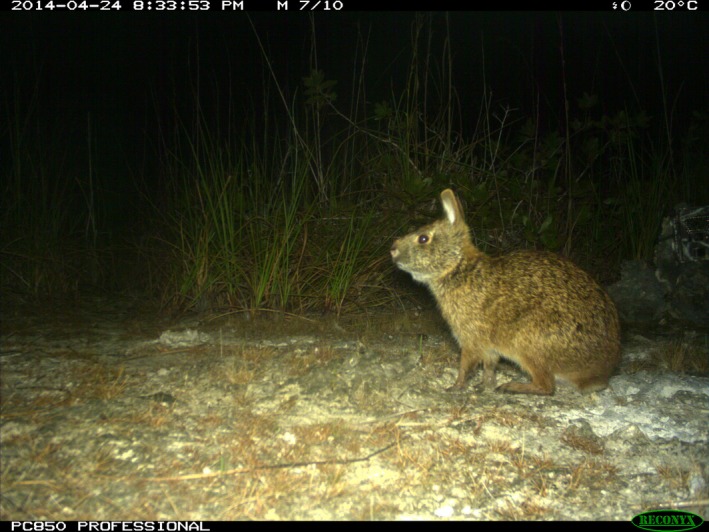
Camera trap image of a Lower Keys marsh rabbit (*Sylvilagus palustris hefneri*) in the National Key Deer Refuge, Big Pine Key, Florida

Eighty percent of historically recorded extinctions have occurred on islands (Ricketts et al., 2005). The Florida Keys are no exception to this pattern with 29 federally protected species affected by sea‐level rise, habitat loss, and invasive species. The Lower Keys marsh rabbit (*Sylvilagus palustris hefneri*, hereafter: marsh rabbit—Figure 1), a distinct population segment of the mainland marsh rabbit endemic to the Lower Florida Keys, is one such protected subspecies (Lazell, 1984; Tursi, Hughes, & Hoffman, 2013). Historically abundant across the Lower Keys, marsh rabbit distribution is currently limited to remnant patches of marshes and coastal transition zones. These patches often are fragmented due to development and habitat succession (Eaton, Hughes, Hines, & Nichols, 2014; Schmidt, McCleery, Lopez, Silvy, & Schmidt, 2010). Furthermore, it has been suggested that marsh rabbits are one of the first mammal species affected by the synergistic effects of human development and rising seas (Schmidt, McCleery, Seavey, Cameron Devitt, & Schmidt, 2012).

Concurrent with expanding anthropogenic development, exotic predators such as large constrictor snakes (e.g., *Python* and *Boa* spp.), tegus (*Salvator merianae*), and feral and free‐ranging domestic cats (*Felis catus*) are now established throughout south Florida and are potential threats to endangered endemic species such as the marsh rabbit in the Keys (Cove, Gardner, Simons, Kays, & O'Connell, 2018; Dorcas et al., 2012; Engeman, Jacobson, Avery, & Meshaka, 2011). McCleery et al. (2015) concluded that recent marsh rabbit declines in south Florida are a result of predation by exotic pythons, in spite of high habitat quality and rabbit fecundity. A recent global analysis of biodiversity loss concluded that exotic mammalian predators are the most detrimental to rare and endangered species, particularly on islands (Doherty, Glen, Nimmo, Ritchie, & Dickman, 2016). Forys (1995) concluded that cats were important marsh rabbit predators based on mortalities of radio‐tagged individuals, but Schmidt et al. (2010) identified raccoons (*Procyon lotor*) as a potential threat using data from track surveys. Furthermore, marsh rabbit population projection models suggested that cat predation was likely the single most important factor hindering marsh rabbit recovery (Forys & Humphrey, 1999; LaFever et al., 2008). These authors concluded that unless cat predation was reduced or eliminated, recovery techniques such as habitat restoration, reintroduction, and the establishment of habitat corridors across the rabbit's range would fail (Forys & Humphrey, 1999).

Free‐ranging domestic cats (feral, colony, and indoor/outdoor house cats) are abundant in the Florida Keys because cats are subsidized by humans and have prodigious reproductive potential (Cove et al., 2018). Evidence of interactions between free‐ranging cats and marsh rabbits is limited and typically anecdotal (i.e., Forys, 1995), or potentially biased because cats can kill marsh rabbits without consuming them (e.g., for stable isotope analyses—Cove et al., 2018). A recently published integrated pest management plan (USFWS, 2013) described protection strategies for endangered species, including the removal of exotic predators, on refuge lands. Indeed, global analyses have suggested that exotic mammal eradications on islands can have strong conservation results for endangered and endemic species, but only three species of mammals have been documented to recover after exotic mammal eradications (Jones et al., 2016b). We used the current exotic predator management regime as an opportunity to examine the effectiveness of predator removal by quantifying the responses of the marsh rabbit population.

Pellet searches are a common survey method to estimate the distribution and abundance of marsh rabbits (Eaton, Hughes, Nichols, Morkill, & Anderson, 2011; Schmidt, McCleery, Schmidt, Silvy, & Lopez, 2011a; Schmidt et al., 2011b), but these methods are rarely validated or tested to meet model assumptions, for example, population closure (Rota, Fletcher, Dorazio, & Betts, 2009). Camera trapping is a valuable survey method because it provides detection/nondetection data to estimate detection probability and spatial and temporal patterns of co‐occurrence among species (Burton et al., 2015; O'Connell, Nichols, & Karanth, 2010). We used camera trapping data to examine factors associated with marsh rabbit occupancy and to determine how habitat and predator–prey dynamics changed during a period of exotic predator removal. Previous experiments revealed that snowshoe hare (*Lepus americanus*) population densities were highest in plots with reduced mammalian predator exposure and added food resources (Krebs et al., 1995), so we predicted similar patterns for marsh rabbit relationships with predators and habitat resources. Specifically, we hypothesized that habitat characteristics, such as freshwater marsh and patch size, would relate positively to marsh rabbit occupancy, but that rabbit occurrence would be negatively related to factors associated with anthropogenic development. We also hypothesized a negative relationship between marsh rabbit occupancy and cat occurrence, and a positive relationship between cat removal and rabbit site colonization rates.

## 2 MATERIALS AND METHODS

### 2.1 Study area

**Figure 2:**
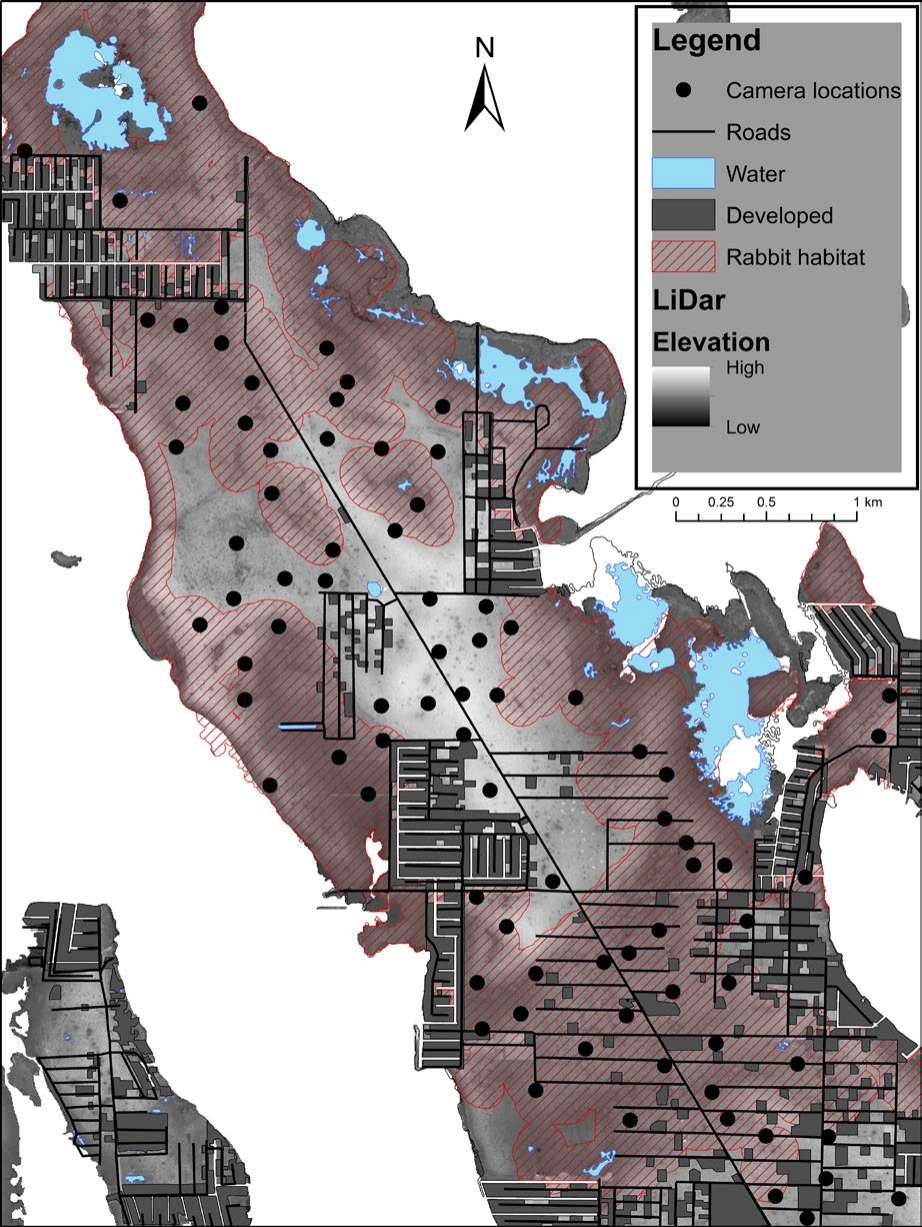
Camera trap locations, habitat, and anthropogenic areas from occupancy surveys of Lower Keys marsh rabbits (*Sylvilagus palustris hefneri*) and free‐ranging domestic cats (*Felis catus*) at the National Key Deer Refuge, Big Pine Key, Florida, 2013–2015

We conducted camera trap surveys on the National Key Deer Refuge, and other public lands managed by the U.S. Fish and Wildlife Service, on Big Pine Key, Florida (Figure 2). The refuge was established in 1957 to protect key deer (*Odocoileus virginianus clavium*) and 20+ other endangered and threatened species including the marsh rabbit. Refuge habitat comprises mostly upland habitats of pine (*Pinus elliottii*) rocklands and tropical hardwood hammocks (e.g., pigeon plum [*Coccoloba diversifolia*], poisonwood [*Metopium toxiferum*], and gumbo‐limbo [*Bursera simaruba*]), whereas lowland habitats, which are more characteristically marsh rabbit habitat, comprise mangroves (e.g., red [*Rhizophora mangle*], black [*Avicennia germinans*], and white [*Laguncularia racemose*]), buttonwood (*Conocarpus erectus*) transition zones, and fresh (*Cladium* spp.) and saltwater (*Spartina* spp.) marshes (USFWS, 1999). The climate is subtropical with mean temperature of 24.63°C ± 4.20*SD* during the transition between the dry and rainy seasons.

Prior to establishment of the refuge, historic rabbit habitat was converted for residential and commercial development, which altered the topography and hydrology of the remaining habitats. Most notably, mosquito ditches were carved into the limestone substrate of Big Pine Key to manage water levels and reduce marshes and standing water. Much of the island was gridded with roads that affect flow and drainage regimes. Restoration efforts for marsh rabbits and other endangered species on the refuge include the removal of roads, prescribed fires in wetlands and pine rocklands to promote native vegetation, and the removal of exotic plants and animals (USFWS, 1999).

### 2.2 Data collection

Our initial sampling design was implemented as part of a free‐ranging cat capture–recapture study (Cove et al., 2018). A 300‐m grid was established to delineate 9‐ha camera sampling units on the refuge and adjacent public (state and county) lands. We sampled 112 camera trap sites from 16 January 2013 through 4 April 2013 using an adaptive sampling approach in which initial camera data were used to inform subsequent sampling to increase the number of detections of both target species (i.e., Cove et al., 2018). We surveyed an additional 84 sites using the same protocol from 16 April 2014 to 2 June 2014. Because those initial surveys were potentially biased toward cat detections due to the adaptive sampling, we selected and resurveyed a total of 84 sites (45 sites from 2013 and 39 sites from 2014) between 28 March 2015 and 14 May 2015 for the dynamic occupancy analyses. We selected these sites because they occurred in suitable marsh rabbit habitat (*n* = 59) or occurred in representative remaining habitat (*n* = 25) in sampling gaps across the island. Therefore, we considered these sites to be unbiased for occupancy estimation of both species because they are representative of the entire wildlife refuge. Cameras were left active at each site for 15–16 days, whereas total sampling occurred during 78 days in 2013 and 47 days in 2014 and 2015. These relatively short sampling periods are comparable to previous occupancy studies applied to camera trap data of small mammals and mesopredators (e.g., Jones et al., 2016a). Marsh rabbit gestation lasts 30–37 days (Chapman & Willner, 1981), and cat gestation lasts 62–71 days (Root, Johnston, & Olson, 1995), with year‐round reproduction for both species, so the primary sampling periods meet the closure assumptions for these target species in dynamic occupancy models (MacKenzie, Nichols, Hines, Knutson, & Franklin, 2003) because births/deaths and immigration/dispersal are unlikely. However, as domestic cats move relatively long distances and we could identify individual cats moving between sites, we considered cat occupancy to more appropriately represent their site use.

Specific camera locations were not randomly selected within grid cells, but were chosen by selecting game trails and natural funnels to ensure high detection rates of rabbits and free‐ranging domestic cats. Each camera site had two opposing camera traps (Reconyx PC800 or PC850, RECONYX, Inc., Holmen WI, USA) set to provide multiple high‐quality photographs so that we could identify a high proportion of photographed animals. Cameras were spaced 2–5 m apart depending on habitat features or trail width.

### 2.3 Statistical methods

Occupancy models commonly use camera trap data to assess individual species distributions, community dynamics, and species interactions (MacKenzie, Bailey, & Nichols, 2004; MacKenzie et al., 2002). Dynamic occupancy models estimate occupancy in the initial sampling period (ψ), colonization (γ), extinction (ε), and detection (*p*) parameters and when used with a co‐occurrence model, they can also estimate conditional probabilities for one species when a second species is present/absent (Bailey, Reid, Forsman, & Nichols, 2009). We created daily detection histories (0 = no detection and 1 = detected) for marsh rabbits and free‐ranging cats at each camera site. We first modeled occupancy of marsh rabbits to determine habitat and predator relationships with dynamic parameters (MacKenzie et al., 2003). We then used dynamic co‐occurrence models to further examine predator–prey relationships by including conditional probabilities of occupancy and species interaction factors (MacKenzie et al., 2004). We used the two modeling approaches because although we expected the results to show similar relationships, we thought it was informative to compare results from contrasting approaches. We used the single‐species models to inform habitat covariates that we included in the co‐occurrence models, to avoid over parameterizing the latter.

We characterized camera sites based on covariates that we identified a priori as potentially important for marsh rabbit and free‐ranging cat distribution and dynamics on Big Pine Key. We identified the habitat type at each camera sampling point as either one of three categorical cover groups: (1) freshwater marshes, (2) coastal marshes, including buttonwood transition zones and scrub mangrove, or (3) upland habitat, including pine and hammock habitats. We also measured habitat patch size and noted whether patches and a 100‐m surrounding buffer were considered marsh rabbit habitat or other habitats (Figure 2). Animal detection probabilities often vary on and off trails, so we also included presence of human trails as a binary covariate if camera stations were located directly on or off trails. We used LiDar data to calculate the mean elevation surrounding each camera trap site because elevation determines the susceptibility of sites to flooding and saltwater incursion from storms and hurricanes. We measured the mean elevation within a 25‐m buffer centered on the cameras because cameras were sometimes located on artificially elevated berms. Human settlements are common throughout Big Pine Key and we suspected that development would affect the distribution of both species, so we measured the linear distance of all camera sites to the nearest residential development. These were all considered habitat covariates in dynamic occupancy models.

The U.S. Fish and Wildlife Service conducted predator removal in marsh rabbit habitats over the course of our surveys. We measured the linear distance between the closest locations where cats were removed in 2014 and 2015 relative to our camera sites and used this measure as a continuous covariate to represent predator removal. We also buffered all sites where cats were trapped in 2014 and 2015 by 500 m and considered any camera sites that fell within those buffers as trapped sites, which we included as a binary covariate in our analyses. The 500 m cutoff was used because previous research suggested that upwards of 90% of the cat population on Big Pine Key move less than that distance in a 2‐month sampling window (Cove et al., 2018). Cats were removed and new cats recruited at sites throughout the course of our study. Therefore, we calculated the change in cat detections (camera trap captures) between the first sampling year and the final sampling year (e.g., negative numbers = relative reduction in cat detections and positive numbers = relative gain in cat detections). We also calculated the relative differences in the number of individual cats detected at each site because cats were easily distinguishable based on pelage (Cove et al., 2018). The change in cat detections and number of individual cats were also representative of predator removal covariates in the subsequent dynamic models. We standardized all continuous covariates to *z*‐scores and used ArcGIS 10.0 (Environmental Systems Research Institute [ESRI], Inc., Redlands, CA, USA) for all spatial measurements and calculations.

We used a multiphase approach to model marsh rabbit occupancy and dynamic parameters, in which we modeled each parameter with the most supported covariate set for the previous parameter starting with detection, then occupancy, colonization, and extinction. We followed a similar approach for modeling the occupancy of free‐ranging cats, but did not model colonization because the parameter of most interest was extinction due to predator removal. Because the first primary survey periods occurred over two seasons, we compared a year‐specific model to a constant model to determine whether we needed to account for variation between 2013 and 2014 sampling in subsequent models. We then modeled detection probability (*p*) as constant across surveys under the global occupancy parameterization. We compared three additional models with marsh rabbit habitat and human trails as single binary covariates and an additive (global) model with both covariates for rabbit detection. We used an information‐theoretic ranking with AIC to determine which detection covariates to include in subsequent occupancy models (Burnham & Anderson, 2002).

We compared 13 occupancy (ψ) hypotheses based on habitat, human disturbance, exotic predators, and combinations of factors (Table S1) and ranked them based on AIC model selection. We then used the most supported occupancy covariate set in all subsequent colonization (γ) models. We included several cat removal covariates to account for changes across sites between years compared to the constant colonization model. We also included patch and habitat effects on colonization (γ) rates to compare with previous studies (Eaton et al., 2014). We compared eight colonization models (Table S1). Finally, we used the most supported colonization covariate set to model extinction (ε) parameters. We examined whether extinction (ε) was affected by patch size or coastal covariates, as well as cat covariates including the number of individual cats at a site or the number of cat detections in the final year (four hypotheses—Table S1). We ranked all models by their relative AIC value and model weights. We considered covariates to have strong effects if they were contained in multiple competing models and their 95% confidence intervals did not overlap zero (Burnham & Anderson, 2002). Additionally, we compared 15 a priori dynamic models of cat site use of camera trap sites following the same framework, excluding any covariates on colonization, and including a year effect based on the first year the site was sampled (e.g., 2013 vs. 2014) because it was supported in the initial candidate comparison (Table S2).

We then modeled co‐occurrence of cats and rabbits over the two primary survey periods to examine interactions and further understand drivers of species extinction and colonization. We used the most influential covariates, as determined by β coefficient 95% confidence intervals that excluded 0, from the previous single‐species models for each species in the subsequent co‐occurrence models. Additionally, we modeled occupancy and detection of rabbits as conditional on cat occurrence and detection at the same sites. Models to estimate the level of species co‐occurrence are structured as:

$$\hat{\varphi }={\hat{\psi }}^{\mathrm{CR}}/{\hat{\psi }}^{C}{\hat{\psi }}^{R}$$

in which ${\hat{\psi }}^{C}$ and ${\hat{\psi }}^{R}$ are the estimated probabilities of site use by cats (C) and rabbits (R), and ${\hat{\psi }}^{\mathrm{CR}}$ is the estimated probability that a site is used by both species. When species co‐occur randomly, the species interaction factor φ = 1, whereas φ < 1 suggests species co‐occur less than randomly expected and φ > 1 suggests species co‐occur greater than randomly expected (MacKenzie et al., 2004). Similarly, the detection interaction factor (δ) is a measure of co‐detection (e.g., greater than randomly expected δ > 1, and less than randomly expected δ < 1) and is conditional on both species being present. We compared eight a priori models to estimate these parameters (Table 3). Occupancy (ψ) was modeled species‐specific in which rabbit occurrence was related to freshwater marsh habitat and patch size and cat occurrence was related to distance to human developments. Species interaction factors (φ) and detection interaction factors (δ) were modeled explicitly or fixed as 1 (e.g., species occur and are detected independently). The probability of detection (*p*) was also modeled species‐specific in which rabbit and cat detection were both related to sites occurring on trails and cat detection additionally varied in rabbit habitat and according to the primary survey period occurring in 2013. Colonization (γ) and extinction (ε) parameters were also species‐specific where cat parameters varied according to the primary survey period occurring in 2013, and rabbit parameters were modeled as constant or conditional on the presence of free‐ranging cat site use. We performed all analyses in the R package “unmarked” and program Presence 11.0 (Fiske & Chandler, 2011; Hines, 2012; R Development Core Team, 2011).

## 3 RESULTS

### 3.1 Camera trap summary statistics

Our camera sampling effort resulted in 2,562 trapdays across 84 sites between January 2013 and May 2015. These surveys resulted in 160 trapdays with marsh rabbit detections, with 58 trapdays (mean = 0.69 detections per site ±1.23*SD*) with detections across sites surveyed in 2013–2014 and 102 trapdays (mean = 1.20 detections per site ±2.00*SD*) with detections at sites surveyed in 2015. There were 270 trapdays with free‐ranging domestic cat detections, with 163 trapdays (mean = 1.9 detections per site ±2.4*SD*) with detections across sites surveyed in 2013–2014 and 107 trapdays (mean = 1.27 detections per site ±2.16*SD*) with detections at sites surveyed in 2015.

### 3.2 Detection from single‐species models

The constant model for initial rabbit occupancy, colonization, extinction, and detection was more supported than including a year effect (ΔAIC = 3.98), so we did not include any covariates to distinguish 2013 from 2014 primary survey periods in further marsh rabbit models. There was support for a primary survey year effect for initial domestic cat occupancy, colonization, extinction, and detection, over the constant model (ΔAIC = 91.53), so we included a binary covariate to distinguish 2013 from 2014 primary survey periods in further free‐ranging cat models.

The top‐supported marsh rabbit detection model with the global occupancy parameterization suggested that marsh rabbit detection probability was negatively related to camera locations on human trails (β = −0.50 ± 0.23*SE*), so we included this covariate in all further occupancy models. The top‐supported free‐ranging cat detection model with the global occupancy parameterization suggested that free‐ranging cat detection probability was negatively related to camera locations on human trails (β = −0.63 ± 0.16*SE*), negatively related to camera locations in marsh rabbit habitat (β = −0.54 ± 0.15*SE*), and negatively related to the primary survey period occurring in 2013 (β = −1.04 ± 0.17*SE*), so we included those covariates in all further free‐ranging cat occupancy models.

### 3.3 Occupancy from single‐species models

Thirteen of the 25 models were contained in the 95% confidence set explaining variation in marsh rabbit occupancy dynamics (Table 1). The global model was most supported to predict initial occupancy, but only three covariates were strong with parameter 95% confidence intervals that excluded 0 (Table 2). Marsh rabbit initial occupancy was positively related to freshwater habitat (β = 3.71 ± 1.53*SE*) and habitat patch size (β = 1.37 ± 0.51*SE*), and negatively related to the number of individual free‐ranging cats identified at each site (β = −1.09 ± 0.45*SE*). Distance to development (+), elevation via LiDar (−), and coastal habitat (−) relationships were not significant but agreed with our a priori predictions. Sites occurring on human trails (+) and within rabbit habitat (−) disagreed with our a priori predictions, but were also not significant.

**Table 1:** Model selection statistics for the top dynamic occupancy models (∑ω > 0.95) with logit‐scale coefficients (β) of habitat and predator management covariates on the probability of site occupancy (ψ), colonization (γ), and extinction (ε) by Lower Keys marsh rabbits (*Sylvilagus palustris hefneri*) from camera trap surveys in the National Key Deer Refuge, Big Pine Key, FL, 2013–2015

				Colonization (*SE*)		Extinction (*SE*)	
Model^a^	Δ_*i*_	ω_*i*_	*K*	β0	β1	β0	β1
[20]ψ(global),γ(ind change),ε(.)	0.00	0.210	14	−4.44 (1.60)	−1.09 (0.60)	−2.09 (0.77)	−
[23]ψ(global),γ(ind change),ε(coastal)	1.43	0.100	15	−4.44 (1.61)	−1.09 (0.60)	−2.20 (0.82)	1.92 (1.97)
[24]ψ(global),γ(ind change),ε(2015 caps)	1.78	0.087	15	−4.45 (1.61)	−1.09 (0.60)	−1.93 (0.79)	−0.23 (0.57)
[22]ψ(global),γ(ind change),ε(patch)	2.00	0.078	15	−4.44 (1.61)	−1.09 (0.60)	−2.11 (0.81)	0.05 (0.80)
[25]ψ(global),γ(ind change),ε(2015 inds)	2.00	0.078	15	−4.44 (1.61)	−1.09 (0.60)	−2.11 (0.91)	0.05 (1.01)
[13]ψ(global),γ(.),ε(.)	2.02	0.077	13	−2.80 (1.02)	—	−2.13 (0.80)	—
[14]ψ(global),γ(dist 2014 trap),ε(.)	2.22	0.070	14	−9.11 (6.50)	−6.27 (5.07)	−2.18 (0.81)	—
[16]ψ(global),γ(2014 trap),ε(.)	2.24	0.069	14	−3.40 (1.20)	1.98 (1.48)	−2.07 (0.76)	—
[15]ψ(global),γ(dist 2015 trap),ε(.)	2.89	0.050	14	−3.05 (1.13)	−0.97 (1.08)	−2.11 (0.78)	—
[18]ψ(global),γ(2014‐15 trap),ε(.)	3.37	0.039	14	−3.21 (1.17)	1.24 (1.48)	−2.09 (0.77)	—
[02]ψ(hab),γ(.),ε(.)	3.45	0.038	12	−3.99 (2.17)	—	−2.23 (0.85)	—
[21]ψ(global),γ(patch),ε(.)	3.61	0.035	14	−2.75 (0.96)	0.40 (0.61)	−2.11 (0.79)	—
[19]ψ(global),γ(cap change),ε(.)	3.63	0.034	14	−3.03 (1.07)	−0.14 (0.21)	−2.12 (0.79)	—

Symbols include Δ_*i*_ is AIC difference, ω_*i*_ is the Akaike weight, and *K* is the number of model parameters. Occupancy (ψ) was modeled under a global parameterization unless otherwise stated.

a Covariate abbreviations: ind change = change in the number of individual cats detected between surveys; coastal = binary covariate differentiating coastal and freshwater sites; 2015 caps = detections of cats at the site in 2015; 2015 inds = number of individual cats detected at the site in 2015; 2014 trap = binary covariate when site was within 500‐m buffer of cat removed in 2014; dist 2014 trap = distance to closest trapped cat in 2014; dist 2015 trap = distance to closest trapped cat in 2015; 2014–2015 trap = binary covariate for sites within 500‐m buffer of trapping locations in 2014 and/or 2015; cap change = change in the number of detections of cats between surveys; patch = size of the patch camera sampled; hab = habitat covariates only.

**Table 2:** Estimated logit‐scale coefficients (β) with standard errors (*SE*), 95% confidence intervals (LCI, UCI), *p* values, and a priori predictions for covariate effects from the top‐ranking dynamic occupancy model explaining variation in initial occupancy and detection of Lower Keys marsh rabbits (*Sylvilagus palustris hefneri*) from camera trap surveys in the National Key Deer Refuge, Big Pine Key, FL, 2013–2015

	β	*SE*	LCI	UCI	*p* value	a priori
Occupancy						
Intercept	−0.80	1.23	−3.21	1.61	.52	
Human trail	2.17	1.15	−0.08	4.43	.06	−
Rabbit habitat	−0.58	1.51	−3.53	2.37	.70	+
Distance to development	0.22	0.42	−0.59	1.04	.59	**+**
Freshwater	**3.71**	**1.53**	**0.72**	**6.70**	**.01**	**+**
Coastal	−1.07	1.67	−4.35	2.22	.52	−
Patch	**1.37**	**0.51**	**0.37**	**2.36**	**.01**	**+**
LiDar	−1.55	0.80	−3.12	0.01	.05	**−**
Individual cats	**−1.09**	**0.45**	**−1.97**	**−0.21**	**.02**	**−**
Detection						
Intercept	−1.70	0.10	−1.90	−1.49	.00	
Human trail	**−0.50**	**0.23**	**−0.94**	**−0.05**	**.03**	**−**

Significant covariate effects and a priori predictions that correspond with estimates are bolded.

Three of the sixteen models were contained in the 95% confidence set explaining variation in free‐ranging domestic cat site use dynamics (Table S3). The global model was most supported to predict initial occupancy, but only the distance from development (β = −1.48 ± 0.60*SE*) was significant with a 95% confidence interval that excluded 0 (Table S4). Nearly all other covariate effects agreed with our a priori predictions: sites located on human trails (+), rabbit habitat (−), freshwater habitat (−), coastal habitat (−), and patch size (−). Elevation via LiDar (−) was the only covariate relationship that disagreed with our a priori predictions.

### 3.4 Colonization and extinction from single‐species models

We observed 10 rabbit colonization events and seven extinction events between the primary survey periods. Five models including the relative change in individual free‐ranging cats detected at each site received more support than the constant colonization model (Table 1). A positive relative change in cats detected at each site (e.g., more individuals in 2015 relative to previous survey years) was negatively related to marsh rabbit patch colonization (β = −1.09 ± 0.60*SE*). The probability of marsh rabbit extinction from individual sites was not strongly explained by any examined covariates (Table 1).

We observed nine free‐ranging cat colonization events and 27 site extinction events between the primary survey periods. The top free‐ranging cat model included a year relationship with extinction. Free‐ranging cat site extinction was positively (β = 2.04 ± 0.67*SE*) related to the time since the primary survey period (e.g., 2 years from 2013 vs. 1 year since 2014—Table S4). The model with free‐ranging cat site extinction varying within a 500‐m buffer of cat trapping areas (−) also received some support, but the relationship disagreed with our a priori hypotheses and was not significant.

### 3.5 Dynamic co‐occurrence results

Four of the eight dynamic co‐occurrence models received support as the 95% confidence set explaining variation in marsh rabbit–cat co‐occurrence dynamics (Table 3). Overall, cats were positively associated with human development ranging from moderate occupancy at sites away from development (ψ^C^ **= 0.57 ± 0.26*SE*) to high (ψ^C^ **= 0.80 ± 0.09*SE*) at sites in close proximity to human development. Marsh rabbit occurrence ranged from very low (ψ^R^ **= 0.07 ± 0.06*SE*) in small coastal and upland habitat patches to high (ψ^R^ **= 0.80 ± 0.11*SE*) in large freshwater marshes (Table 3). The top‐supported model suggested that rabbit site extinction probability varied between sites occupied by free‐ranging cats (ε = 0.19 ± 0.14*SE*) compared to sites without cats (ε = 0.02 ± 0.14*SE*), but the confidence intervals strongly overlapped. Free‐ranging cat site extinction probability varied according to the time since the primary survey period with extinction probability higher at sites with 2 years of predator trapping (ε = 0.70 ± 0.11*SE*) versus sites with only 1 year of predator trapping (ε = 0.20 ± 0.09*SE*). The species interaction factor from the top‐supported model (φ = 0.81 ± 0.15*SE*) was less than 1, suggesting the two species co‐occurred less frequently than random, but the 95% confidence interval slightly overlapped with 1. Additionally, two of the four top‐supported models contained φ = 1 as a fixed parameter. The detection interaction factor varied significantly and was lower than 1 at sites first surveyed in the 2013 primary survey period (δ = 0.53 ± 0.16*SE*) and was higher than 1 (δ = 3.55 ± 0.95*SE*) at sites first surveyed in the 2014 primary survey period (Table 3).

**Table 3:** Model selection statistics, parameter estimates (±*SE*), and their ranges across surveyed sites if applicable for dynamic multispecies models for co‐occurring free‐ranging cats (C) and Lower Keys marsh rabbits (R) in the National Key Deer Refuge, Big Pine Key, Florida, surveyed 2013–2015

Model^a^	Δ_*i*_	ω_*i*_	*K*	ψ^C^	ψ^R^	ϕ	δ
**ψ(sp),ϕ,γ(sp),ε(pr),p(sp),δ**	0	0.345	21	0.57 (0.26) 0.80 (0.09)	0.07 (0.06) 0.80 (0.11)	0.81 (0.15)	0.53 (0.16) 3.55 (0.95)
**ψ(sp),γ(sp),ε(sp),p(sp),δ**	0.13	0.323	19	0.46 (0.22) 0.87 (0.06)	0.08 (0.05) 0.89 (0.08)	1	0.56 (0.18) 3.55 (1.06)
**ψ(sp),ϕ,γ(pr),ε(sp),p(sp),δ**	0.97	0.212	21	0.56 (0.24) 0.80 (0.09)	0.07 (0.06) 0.80 (0.11)	0.82 (0.15)	0.54 (0.17) 3.46 (0.95)
**ψ(sp),γ(sp),ε(sp),p(sp)**	3.35	0.065	18	0.46 (0.21) 0.86 (0.06)	0.08 (0.05) 0.88 (0.07)	1	1
ψ(sp),γ(pr),ε(sp),p(sp)	5.12	0.027	19	0.46 (0.21) 0.86 (0.06)	0.08 (0.05) 0.88 (0.07)	1	1
ψ(sp),γ(sp),ε(pr),p(sp)	5.22	0.025	19	0.46 (0.21) 0.86 (0.06)	0.08 (0.05) 0.88 (0.08)	1	1
ψ(sp),ϕ,γ(pr),ε(pr),p(sp),δ	9.63	0.003	22	0.55 (0.25) 0.85 (0.08)	0.08 (0.05) 0.84 (0.09)	0.90 (0.10)	0.87 (0.19) 5.24 (0.00)
ψ(sp),ϕ,γ(sp),ε(sp),p(sp)	15.1	0.000	19	0.50 (0.23) 0.81 (0.10)	0.07 (0.05) 0.83 (0.13)	0.86 (0.18)	1

Bolded models make up the 95% confidence set (∑ω > 0.95).

a Occupancy (ψ) was modeled species‐specific (sp) in which rabbit occurrence was related to freshwater marsh habitat and patch size and cat occurrence was related to distance from human development. Species interaction factors (φ) and detection interaction factors (δ) were modeled explicitly or fixed as 1 (e.g., species occur and are detected independently); probability of detection (*p*) was modeled species‐specific (sp) in which rabbit and cat detection were both related to sites occurring on trails and cat detection additionally varied according to the primary survey period occurring in 2013 and in rabbit habitat. Colonization (γ) and extinction (ε) parameters were also species‐specific where cat parameters varied according to the primary survey period occurring in 2013 and rabbit parameters were modeled as constant (sp) or conditional on the presence of predators (pr); Δ_*i*_ is the information distance from the top‐ranked model, ω_*i*_ is the Akaike weight, and K is the number of parameters.

## 4 DISCUSSION

### 4.1 Marsh rabbit habitat relationships

Marsh rabbit occupancy was strongly related to habitat features including freshwater marshes and patch size. In a study based on rabbit pellet counts, Eaton et al. (2011) observed that interior freshwater marshes were more important refugia for marsh rabbits than coastal patches. However, Schmidt et al. (2011b) conducted similar pellet‐based surveys and concluded that salt‐tolerant habitats such as mangroves and transition zones were beneficial to marsh rabbits after Hurricane Wilma. We suspect that while salt‐tolerant coastal habitats are more resilient to hurricane storm surges, our data support the notion that freshwater wetlands are valuable as marsh rabbit refugia on Big Pine Key. We sampled sites in the late dry and early transition into the rainy season, so most of the freshwater marshes were accessible. These areas flood during the rainy season and might have further value as refugia from potential predators that do not cross open water. Cats can swim (Abbott, 2000), but we suspect they would not be inclined to cross water barriers unless resources (e.g., human‐provisioned food) were limited. Marsh rabbit occupancy was also positively related to patch size, which corresponds with both recent studies (Eaton et al., 2011; Schmidt et al., 2011b) and historical surveys (Forys, 1995). These results suggest that restoration efforts, particularly the removal of old roads and berms that affect flow regimes and fragment marsh patches, will be most beneficial to marsh rabbits if they produce large contiguous freshwater wetlands.

### 4.2 Marsh rabbits and co‐occurring free‐ranging domestic cats

In addition to habitat covariates, marsh rabbit occupancy was related to indicators of predator presence and abundance. The top model suggested that the number of individual cats detected at sites had a strong negative relationship with initial rabbit occupancy, supporting the hypothesis that free‐ranging domestic cats play some role in the distribution of endangered marsh rabbits on Big Pine Key. Additionally, dynamic co‐occurrence models suggested cats and marsh rabbits co‐occur less frequently than expected under random conditions, but the 95% confidence interval included 1 (e.g., random expectation). These varying results reveal that cat abundance is a better predictor of marsh rabbit occurrence than cat presence alone. Prior to management, cat densities on Big Pine Key were high (>4 cats/km^2^) and the home ranges of individual cats varied according to their ownership status (Cove et al., 2018). Cat movement typically overlapped several survey locations and marsh rabbit home ranges (e.g., 3.96 ha ±0.65*SE*—Forys & Humphrey, 1996). As many as seven individual cats visited a single camera trapping site, suggesting that the potential for interactions with marsh rabbits also increases with cat abundance. Presumably trapping individual cats that range long distances reduces predation pressure in marsh rabbit habitat elsewhere on the island.

The U.S. Fish and Wildlife Service trapped and removed approximately 40 free‐ranging cats from the refuge between the primary camera sampling periods of our study. This removal effort was substantial considering 2013 camera trap surveys only identified 54 individuals on the refuge and density estimates suggested an island‐wide abundance of 120+ individual cats (Cove et al., 2018). We observed cat site extinction events at three times as many sites as cat colonization events, and site extinction was related to the longer 2‐year trapping period between survey years (e.g., sites first surveyed in 2013). These results suggest that the trapping effort was most likely responsible for the observed differences in individual cats detected at sites between years. The negative relationship between the change in the number of individual cats detected at each site, and rabbit colonization of those sites, provides further support for the hypothesis that cats reduce marsh rabbit abundance. Cat eradication on Big Pine Key is not likely possible because many cats on the island are owned or associated with human developments and because cat eradication is difficult (Nogales et al., 2004). Nevertheless, our results indicate that reductions in the number of individual cats are an effective management practice that promotes marsh rabbit colonization of vacant habitats.

### 4.3 Future research

When both rabbits and cats occur at a site, the co‐detection interaction factors varied according to site‐specific covariates. In particular, sites surveyed in 2013 suggested co‐detection to be less than randomly expected (δ < 1), whereas sites surveyed in 2014 suggested co‐detection to be greater than randomly expected (δ > 1). This result warrants further study because it could signify some seasonal variation in cat movement and hence detection that we did not consider in the survey design, where cats are more detectable in early spring compared to late winter. This result could have biologically relevant implications because regardless of the time of year if co‐detections are higher than randomly expected it is probable that direct interactions will also increase. Our dynamic occupancy models for free‐ranging cats and co‐occurrence models suggest that cats occupy a greater range of habitat types on Big Pine Key than marsh rabbits. This is potentially biased because our original survey objective was to conduct spatial capture–recapture to estimate population densities of free‐ranging cats throughout the National Key Deer Refuge, hence the adaptive sampling approach outlined in the methods. However, we suggest that the subsampling of those sites and including additional sites from a survey in 2014 reduced any bias toward free‐ranging cats for the dynamic occupancy modeling approach from the resurvey in 2015. Additionally, sites were representative of the available habitat and distributed throughout the island, so these point samples represent the true proportion of area occupied by cats (Efford & Dawson, 2012). Therefore, the results that suggest cat occurrence was strongly associated with human development but not influenced by any other covariates confirms this species is highly adaptable in developed island environments (Medina et al., 2011). Future research might benefit from more explicitly estimating the effects of potential variation in predator density and home‐range variation and its relationship with prey occupancy at survey sites, across multiple scales.

Previous research examining marsh rabbit distribution and dynamics was based on pellet surveys or radio telemetry of individual rabbits (Eaton et al., 2011; Forys, 1995; Schmidt et al., 2010), but these methods have limitations compared to our camera trap surveys. Rabbit pellets can last in the environment for long periods and potentially inflate estimates of the number of occupied sites. Telemetry studies are also useful, but we suggest that future approaches would benefit from concurrent tracking of both species. The camera trapping protocol that we used provided useful data on predators and their distribution for inferences in co‐occurrence models. Additional sampling with camera traps on other islands could further help clarify the role of predator–prey dynamics affecting the distribution of marsh rabbits throughout the Lower Keys. Long‐term monitoring and careful planning of habitat manipulations could provide further information about marsh rabbit population responses to bottom‐up and top‐down interactions like those observed in snowshoe hares (Krebs et al., 1995).

## 5 CONCLUSION

Habitat is clearly a limiting factor for the Lower Keys marsh rabbit. Large freshwater wetlands provide refugia for the species, but these habitats are highly altered and susceptible to saltwater intrusion due to hurricane tidal surges and sea‐level rise (Eaton et al., 2014; Schmidt et al., 2011b). Current management strategies that eliminate old roads and allow mosquito ditches to fill with sediment will help restore these habitats and their hydrology, both of which will reduce saltwater retention following storm surges. Additionally, the U.S. Fish and Wildlife Service has actively managed exotic predators by removing them from the system. Our results indicate that this approach has helped to reduce the number of individual cats at sites colonized by rabbits since our initial surveys. Simultaneous management of both habitat and exotic predator populations is likely necessary for the recovery of many endangered island endemics, particularly where public lands juxtapose urban landscapes.

## CONFLICT OF INTEREST

None declared.

## AUTHOR CONTRIBUTION

MVC, BG, TRS, and AFO conceived and designed the research; MVC conducted fieldwork and collected data; MVC analyzed the data; TRS, BG, and AFO contributed materials/analysis tools and consulted on analyses; MVC wrote the initial draft of the manuscript; MVC, TRS, BG, and AFO revised and edited the manuscript.
