# Supplementary material for: Co‐occurrence dynamics of endangered Lower Keys marsh rabbits and free‐ranging domestic cats: Prey responses to an exotic predator removal program
Source: Ecol Evol. 2018 Mar 26;8(8):4042–52. doi: 10.1002/ece3.3954 (PMC5916284; doi:10.1002/ece3.3954)
Supplement: Supplementary file 1 [file ECE3-8-4042-s001.docx]

| Table S1. Descriptions of *a priori* models (predicted covariate relationship directions in parentheses) for dynamic occupancy models predicting the distribution of the Lower Keys marsh rabbit (*Sylvilagus palustris hefneri*) from camera trap surveys in the National Key Deer Refuge, Big Pine Key, FL, 2013-2015. In each model, detection covariates included distance to human trails and rabbit habitat. Model structure follows the form of the R Package ‘unmarked’ with the ‘colext’ command for dynamic occupancy models. | |
| --- | --- |
| Hypothesis | Model Structure |
| **Constant Model** |  |
| [1] No effects on any parameters | *ψ* (.), γ (.), ε (.),  *p* (humtrail) |
| **Habitat Models** |  |
| [2] Global habitat covariates affect occupancy | *ψ* (humtrail+rabbitat+devel+fresh+salt_butt+patch+LiDar), γ (.), ε (.),  *p* (humtrail+rabbitat) |
| [3] Rabbit habitat affects occupancy (+) | *ψ* (rabbitat), γ (.), ε (.),  *p* (humtrail) |
| [4] Human trails affect occupancy (-) | *ψ* (humtrail), γ (.), ε (.),  *p* (humtrail) |
| [5] Distance to development affects occupancy (+) | *ψ* (devel), γ (.), ε (.),  *p* (humtrail) |
| [6] Freshwater affects occupancy (+) | *ψ* (fresh), γ (.), ε (.),  *p* (humtrail) |
| [7] Patch size affects occupancy (+) | *ψ* (patch), γ (.), ε (.),  *p* (humtrail) |
| [8] Elevation affect occupancy (-) | *ψ* (LiDar), γ (.), ε (.),  *p* (humtrail) |
| **Human Disturbance Model** |  |
| [9] Human disturbances affect occupancy | *ψ* (humtrail+road+devel+patch), γ (.), ε (.),  *p* (humtrail) |
| **Predator Models** |  |
| [10] Cat detections at a site affect occupancy (-) | *ψ* (capsyear1), γ (.), ε (.),  *p* (humtrail) |
| [11] Individual cats at a site affect occupancy (-) | *ψ* (indsyear1), γ (.), ε (.),  *p* (humtrail) |
| [12] Global predator covariates affect occupancy | *ψ* (humtrail+devel+indsyear1), γ (.), ε (.),  *p* (humtrail) |
| **Global Occupancy Model** |  |
| [13] Global habitat and individual cats affect occupancy | *ψ* (humtrail+rabbitat+ devel+fresh+salt_butt+patch+LiDar+indsyear1), γ (.), ε (.),  *p* (humtrail) |
| **Colonization Models** |  |
| [14] Distance to cats trapped in 2014 affects colonization (-) | *ψ* (top occupancy model), γ (cat2014), ε (.),  *p* (humtrail) |
| [15] Distance to cats trapped in 2015 affects colonization (-) | *ψ* (top occupancy model), γ (cat2015), ε (.),  *p* (humtrail) |
| [16] Cats trapped in 2014 in proximity to sites affects colonization (+) | *ψ* (top occupancy model), γ (bin_2014cat), ε (.),  *p* (humtrail) |
| [17] Cats trapped in 2015 in proximity to sites affects colonization (+) | *ψ* (top occupancy model), γ (bin_2015cat), ε (.),  *p* (humtrail) |
| [18] Cats trapped in 2014 and/or 2015 in proximity to sites affects colonization (+) | *ψ* (top occupancy model), γ (bin_20142015), ε (.),  *p* (humtrail) |
| [19] Relative change in cat detections at a site affects colonization (-) | *ψ* (top occupancy model), γ (cap_change), ε (.),  *p* (humtrail) |
| [20] Relative change in individual cats at a site affects colonization (-) | *ψ* (top occupancy model), γ (ind_change), ε (.),  *p* (humtrail) |
| [21] Patch size affects colonization (+) | *ψ* (top occupancy model), γ (patch), ε (.),  *p* (humtrail) |
| **Extinction Models** |  |
| [22] Patch size affects extinction (-) | *ψ* (top occupancy model), γ (top colonization model), ε (patch),  *p* (humtrail) |
| [23] Coastal habitats affect extinction (+) | *ψ* (top occupancy model), γ (top colonization model), ε (salt_butt),  *p* (humtrail) |
| [24] Cat detections in the final year affect extinction (+) | *ψ* (top occupancy model), γ (top colonization model), ε (catcaps),  *p* (humtrail) |
| [25] Cat individuals in the final year affect extinction (+) | *ψ* (top occupancy model), γ (top colonization model), ε (catinds),  *p* (humtrail) |

| Table S2. Descriptions of *a priori* models (predicted covariate relationship directions in parentheses) for dynamic occupancy models predicting the distribution of free-ranging domestic cats (*Felis catus*) from camera trap surveys in the National Key Deer Refuge, Big Pine Key, FL, 2013-2015. In each model, detection covariates included site location on human trails, site location in rabbit habitat, and the year of the primary sampling period (2013 versus 2014). Model structure follows the form of the R Package ‘unmarked’ with the ‘colext’ command for dynamic occupancy models. | |
| --- | --- |
| Hypothesis | Model Structure |
| **Constant Model** |  |
| [1] No effects on any parameters | *ψ* (.), γ (.), ε (.),  *p* (humtrail+rabbitat+year) |
| **Habitat Models** |  |
| [2] Global habitat covariates affect occupancy | *ψ* (humtrail+rabbitat+devel+fresh+salt_butt+patch+LiDar), γ (.), ε (.),  *p* (humtrail+rabbitat+year) |
| [3] Rabbit habitat affects occupancy (-) | *ψ* (rabbitat), γ (.), ε (.),  *p* (humtrail+rabbitat+year) |
| [4] Human trails affect occupancy (+) | *ψ* (humtrail), γ (.), ε (.),  *p* (humtrail+rabbitat+year) |
| [5] Distance away from development affects occupancy (-) | *ψ* (devel), γ (.), ε (.),  *p* (humtrail+rabbitat+year) |
| [6] Freshwater affects occupancy (-) | *ψ* (fresh), γ (.), ε (.),  *p* (humtrail+rabbitat+year) |
| [7] Patch size affects occupancy (-) | *ψ* (patch), γ (.), ε (.),  *p* (humtrail+rabbitat+year) |
| [8] Elevation affects occupancy (+) | *ψ* (LiDar), γ (.), ε (.),  *p* (humtrail+rabbitat+year) |
| **Human Disturbance Model** |  |
| [9] Human disturbances affect occupancy (+) | *ψ* (humtrail +devel+patch), γ (.), ε (.),  *p* (humtrail+rabbitat+year) |
| **Extinction Models** |  |
| [10] Distance away from closest cat trapped in 2014 affects extinction (-) | *ψ* (top occupancy model), γ (.), ε (cat2014),  *p* (humtrail+rabbitat+year) |
| [11] Distance away from closest cat trapped in 2015 affects extinction (-) | *ψ* (top occupancy model), γ (.), ε (cat2015),  *p* (humtrail+rabbitat+year) |
| [12] Cat trapping in 2014 within a 500 m buffer of the site affects extinction (+) | *ψ* (top occupancy model), γ (.), ε (bin_2014cat),  *p* (humtrail+rabbitat+year) |
| [13] Cat trapping in 2015 within a 500 m buffer of the site affects extinction (+) | *ψ* (top occupancy model), γ (.), ε (bin_2015cat),  *p* (humtrail+rabbitat+year) |
| [14] Cat trapping in 2014 & 2015 within a 500 m buffer of the site affects extinction (+) | *ψ* (top occupancy model), γ (.), ε (bin_20142015),  *p* (humtrail+rabbitat+year) |
| [15] Difference of the primary survey period starting year affects extinction (+) | *ψ* (top occupancy model), γ (.), ε (year),  *p* (humtrail+rabbitat+year) |

| **Table S3** Model selection statistics for the top dynamic occupancy models (∑*ω* > 0.95) with logit-scale coefficients (β) of habitat and predator management covariates on the probability of extinction (ε) by free-ranging domestic cats (*Felis catus*) from camera trap surveys in the National Key Deer Refuge, Big Pine Key, FL, 2013-2015. Symbols include Δ_i_ is AIC difference, *ω_i_* is the Akaike weight, and K is the number of model parameters. Coefficients are in logit space and relate to standardized covariate values. Occupancy (ψ) and detection (*p*) were modeled under global models unless otherwise stated. | | | | | | | | | |
| --- | --- | --- | --- | --- | --- | --- | --- | --- | --- |
|  | |  | |  | |  | | Extinction (SE) | |
| Model* | | Δ*_i_* | | *ω_i_* | | *K* | | β0 | β1 |
| [15]ψ(global),γ(.),ε(year) | 0.00 | | 0.913 | | 15 | | −1.21 (0.48) | | 2.04 (0.67) |
| [13]ψ(global),γ(.),ε(2015 trap) | 7.89 | | 0.018 | | 15 | | −0.11 (0.35) | | −1.26 (0.94) |
| [02]ψ(global),γ(.),ε(.) | 8.26 | | 0.015 | | 14 | | −0.36 (0.32) | | − |
| *Covariate abbreviations: year = binary covariate for the difference between primary sampling periods in 2013 versus 2014; 2015 trap = binary covariate when site was within 500m buffer of cat removed in 2015. | | | | | | | | | |

| **Table S4** Estimated beta coefficients (β) with standard errors (SE), 95% confidence intervals (LCI, UCI), and p values for covariate effects from the top-ranking dynamic occupancy model explaining variation in initial occupancy and detection of free-ranging domestic cats (*Felis catus*) from camera trap surveys in the National Key Deer Refuge, Big Pine Key, FL, 2013-2015. Significant covariate effects and *a priori* predictions that correspond with estimates are bolded. | | | | | | |
| --- | --- | --- | --- | --- | --- | --- |
|  | **β** | **SE** | **LCI** | **UCI** | **p value** | ***a priori*** |
| **Occupancy** |  |  |  |  |  |  |
| Intercept | 3.33 | 1.44 | 0.50 | 6.15 | 0.02 |  |
| Human trail | 11.60 | 30.31 | -47.81 | 71.02 | 0.70 | + |
| Rabbit habitat | -2.46 | 1.48 | -5.36 | 0.44 | 0.10 | − |
| **Distance to development** | **-1.48** | **0.60** | **-2.66** | **-0.29** | **0.01** | − |
| Freshwater | -1.61 | 1.44 | -4.43 | 1.21 | 0.26 | − |
| Coastal | -1.43 | 2.24 | -5.83 | 2.97 | 0.52 | − |
| Patch | -0.47 | 0.49 | -1.43 | 0.49 | 0.34 | − |
| LiDar | -0.38 | 0.68 | -1.72 | 0.96 | 0.58 | + |
| **Detection** |  |  |  |  |  |  |
| Intercept | -0.69 | 0.14 | -0.96 | -0.42 | 0.00 |  |
| **Human trail** | **-0.63** | **0.16** | **-0.94** | **-0.31** | **0.00** | + |
| **Rabbit habitat** | **-0.54** | **0.15** | **-0.83** | **-0.25** | **0.00** | **−** |
| **Year** | **-1.04** | **0.17** | **-1.38** | **-0.71** | **0.00** | **−** |
